# Linking Learning Contexts: The Relationship between Students’ Civic and Political Experiences and Their Self-Regulation in School

**DOI:** 10.3389/fpsyg.2016.00575

**Published:** 2016-04-25

**Authors:** Carla Malafaia, Pedro M. Teixeira, Tiago Neves, Isabel Menezes

**Affiliations:** ^1^Centre for Research and Intervention in Education (CIIE), Faculty of Psychology and Educational Sciences, University of PortoPorto, Portugal; ^2^3B’s Associated Laboratory, Life and Health Sciences Research Institute (ICVS), School of Health Sciences, Universidade do Minho, Campus de GualtarBraga, Portugal

**Keywords:** civic and political participation, quality of participation, metacognition, adolescent

## Abstract

This paper considers the relationship between self-regulation strategies and youth civic and political experiences, assuming that out-of-school learning can foster metacognition. The study is based on a sample of 732 Portuguese students from grades 8 and 11. Results show that the quality of civic and political participation experiences, together with academic self-efficacy, are significant predictors of young people’s self-regulation, particularly regarding cognitive and metacognitive strategies (elaboration and critical thinking). Such effects surpass even the weight of family cultural and school variables, such as the sense of school belonging. Therefore, we argue that the pedagogical value of non-formal civic and political experiences is related to learning in formal pedagogical contexts. This is because civic and political participation with high developmental quality can stimulate higher-order cognitive engagement and, thus, contribute to the development of learning strategies that promote academic success.

## Introduction

Scholars and policy makers alike have been discussing the importance of innovative learning and teaching methods in order to prevent early school leaving and prepare future adults to face adverse social contexts (Ainley, 2011; OECD, 2011). Also, at the higher education level, there is an increasing demand for learning models based on learners’ autonomy and independency, as well as on their ability to actively construct their own learning processes (Vermunt, 1996). These, together with high levels of qualification, are regarded as vital assets for preserving European welfare (European Commission, 2011; OECD, 2011).

Pedagogical experiences take place in a variety of contexts that are permeable to each other. Together, they contribute to the construction of the individual’s world view, his/her perceived individual and collective influence, and his/her recognition of the possibilities and conditions to make choices. When students are capable of consciously controlling their learning processes, they acquire knowledge in personally meaningful ways and are therefore better able to achieve superior academic results (Kruger and Dunning, 1999; Zimmerman and Schunk, 2011). It is important, then, to understand the phenomenon of learning regulation, which encompasses specific mental models of learning, learning orientations, cognitive processing, and metacognitive regulation strategies (Vermunt, 1996). Thus, adequate instructional strategies and learning models are crucial in promoting school attainment. Additionally, youth life contexts beyond the school are known to play a relevant role in promoting learning competencies (Ainley, 2011). It is now clear that educational variables are intrinsically connected with civic and political participation (Hadjar and Beck, 2010), and that school education impacts civic and political action and sophistication (Quintelier, 2010; Stockemer, 2014). Pleas are then made for an articulation between the school and society, in which instructional design enhances democratic citizenship, contributing “to the ability as well as the readiness of students to participate independently in a meaningful and critical way in concrete real social practices and activities” (ten Dam and Volman, 2004, p. 371). Also, the pedagogical value of such civic, participatory experiences needs to be recognized as they entail learning processes that improve higher-order skills. However, further research is necessary in order to bridge important research gaps.

So far, research has shown that students’ participation in extra and co-curricular activities has a positive effect on their academic success (e.g., Mezuk et al., 2011; Roberts, 2007, Unpublished). Likewise, the students’ ability to self-regulate their study through the development of metacognitive competencies has a positive effect on their academic success (e.g., Rani and Govil, 2013). Kolb’s learning models highlight the role of learner in his/her learning process, stressing the importance of concrete, contextualized experiences and of reflecting about them in the active construction of knowledge (Kolb, 1984; Demirbas and Demirkan, 2007). Additionally, several studies indicate that individuals with higher educational levels tend to be more civically and politically engaged (e.g., Nie et al., 1996; Rosenstone and Hansen, 2003). Lamm (2009) argues that a link needs to be promoted between students’ metacognitive reflection and political engagement, because critical thinking is crucial to increasing their ability to relate their skills to the real world, by reflecting on their learning experience. In other words, she argues that self-regulated learning is important for civic and political engagement.

Yet, civic and political participation experiences are not to be regarded as eminently or ‘naturally’ positive processes, but rather as experiences that, if they bring about developmental change, can be deemed pedagogical. Developmental quality of participation refers to a combination of features of experiences that take place in meaningful civic and political contexts; this combination is transformative, that is, it promotes more complex modes of comprehension and action (Ferreira et al., 2012). For a participation experience to be qualified as having high developmental quality, the following components need to be present: interaction with different points of view (as the integration of diversity and pluralism promotes cognitive development); reflection about one’ own perspective and participation in such a compelling and challenging environment; action in and reflection on real, meaningful issues (involving personal implication and commitment; Ibidem.). The transformative potential of action and reflection depends, then, on their complementarity, as well as on the duration of the experience, given that development requires long-term continuity. The potential of this approach is the focus on understanding the developmental quality of participation experiences within contexts not necessarily planned to promote developmental transformation.

This study seeks to explain the relationship between civic and political participation experiences and academic performance. We do so, by:

(a) Considering not only the experiences of participation *per se* but the developmental quality associated with such experiences, and
(b) Assessing academic performance through variables that consider the learning process in a more holistic and dynamic way, specifically through dimensions of self-regulated learning.

Therefore, we suggest sketching an innovative relationship, articulating learning processes that take place in different spheres of the students’ lives (inside and outside school). We argue, then, that not only education leads to political knowledge and civic mobilization, but also that civic and political participation can result in educational gains – an approach that is under-researched.

### Metacognition and School Performance

Learning is an active and constructive process, in which the learner plays an important role in building his/her own learning experience (Duffy and Jonassen, 1992), appropriating knowledge in a dynamic way (Bretz, 2001). Metacognition, the ability to think about thinking, is central in this process as it involves an awareness of the cognitive process and ability to control it (Flavell, 1976, 1987). Metacognition has two main components: knowledge about cognition and control/regulation of cognition (Baker and Brown, 1984; Applegate et al., 1994). Knowledge about cognition is related with knowledge about oneself as a learner, considering the characteristics of the task at hand and the strategies available to deal with it, as well as the strategy to apply at a given moment. The control or regulation of cognition concerns the ability to actively plan and evaluate the strategies and skills necessary to approach a specific task, planning the best way to do it, and the skill to reformulate the ongoing process (Baker, 2008). Both cognitive processing and metacognitive regulation lead to important learning results (Brown, 1987; Geisler-Brenstein et al., 1996).

Research on the relationship between metacognition and academic performance identifies its potential for learning enhancement, showing that students with higher levels of metacognition have above average school results (Rani and Govil, 2013; Coutinho, 2006, Unpublished). Some elements associated with metacognition are the ability to link concepts, promoting in-depth questioning and understanding (Stuever, 2006, Unpublished), the use of constructive processing strategies (Vermunt, 1998), the transferability and durability of learning (Bransford et al., 2000), task persistence, the competence to overcome frustrations and the regular exhibition of a sense of self-efficacy (Zimmerman and Schunk, 2011). Furthermore, there is reciprocity between metacognitive awareness and control and learning improvement because they feed each other (Baker, 2008).

### The Relationship between School Performance and Civic and Political Participation

Many studies articulate civic and political participation and school performance. In fact, the school is often regarded as an important element in redressing the worries about youth civic and political participation. This is a topic of social concern, as participation is a crucial feature of democracies. Schools are often considered spaces of political socialization due to their role in potentiating exposure to political messages and providing opportunities for democratic education in practice (Torney-Purta, 2002). Research on the relationship between the school and civic and political participation has been mostly grounded on two research strands: the effects of schooling on civic and political participation (Quintelier, 2010); and the impact of participation in schools, on students’ academic performance (Dávila and Mora, 2007; Mezuk et al., 2011).

Schools can promote students’ political participation by providing a stimulating environment for political discussion, fostering skills for understanding politics, and increasing levels of political interest and attention (Gibson and Levine, 2003; Jennings et al., 2009). Furthermore, the political science literature consistently underscores the intimate relationship between the level of education and the likelihood of participation (Verba et al., 1995; Nie et al., 1996; Rosenstone and Hansen, 2003). Thus, schools are proclaimed as vehicles for learning democratic skills and stimulating political involvement by promoting active learning strategies that involve the discussion of political topics (Regnerus, 2000), fostering engagement in school councils and “real life” activities (Regnerus, 2000; Gibson and Levine, 2003), and creating a participative school culture (Torney-Purta, 2002).

Concerning the second research strand, some literature highlights how civic and political experiences can foster school achievement. Dávila and Mora (2007) analyze the relationship between participation in student government and community service on educational progress after grade 8, indicating that civic engagement promotes academic progress, increasing the odds of remaining in school and actually graduating from college. This study stands out as an important contribution to research focused on the flip side of the coin: that is, on showing that civic activities are a catalyst for educational attainment, as “civically engaged teenagers seemingly acquire higher levels of education on the average than their otherwise similar peers as they get older” (p. 16). Likewise, participation in extra-curricular activities (e.g., urban debate programs) bolsters academic attainment and progress, addressing achievement gaps for low-income and minority students (Mezuk et al., 2011), with some evidence that this is a long-term-effect (Roberts, 2007, Unpublished). Although extra-curricular activities are sometimes pointed out as taking time away from study, many researchers claim that these activities can actually improve the academic achievement of students, increasing their concentration, motivation and aspirations (Khan et al., 2012), as well as boosting their sense of school belonging (Mahoney, 2000), school interest, self-discipline, and academic self-efficacy (Marsh and Kleitman, 2002; Adeyemo, 2010).

### Toward a Disciplinary Bridging: Pedagogical Experiences in Formal and Non-formal Educational Contexts

Youth experiences in schools and in civic and political contexts share a common, important goal: to provide youngsters the tools and opportunities to claim and establish their own place in society. The role of the school in fostering civic competencies is frequently emphasized, along with the idea, advocated by classic and current authors, that active and democratic citizenship should be experienced in relevant life contexts, creating opportunities to think and act (Dewey, 1916; Lawy and Biesta, 2006). An effective instruction process requires the promotion of students’ involvement in school curricula and activities in a meaningful way, providing vast and diverse experiences (Dewey, 1938; Lawy and Biesta, 2006), supporting students’ active engagement in their own learning process (Freire, 1985).

Classical authors in educational theory and psychological development pointed out key elements that promote the pedagogical character of learning experiences. Dewey (1916), Mead (1934), and Piaget (1941) have all stressed the importance of recognizing difference in self-development processes, since it promotes cognitive conflict, and therefore boosts psychological development. This Piagetian notion of ‘reflective abstraction’ can be found in other studies (Kohlberg, 1976; Lind, 2000) that also emphasize the opportunities provided by social interaction as sources of cognitive conflict. The dialogical contact with different perspectives within a challenging and supportive environment promotes cognitive-developmental change (Sprinthall, 1980). More recently, research on civic and political participation investigated their pedagogical value using the same criteria, particularly the potential for promoting opportunities for action and reflection (Menezes, 2003; Ferreira et al., 2012).

The effort of bridging the experiences in non-formal educational contexts and those inside schools entails great heuristic potential. This study seeks to move forward by articulating the pedagogical quality with the learning potential of formal and non-formal educational experiences. Our argument is that formal and non-formal pedagogical experiences are organized along a continuum and can both contribute to metacognition competences. Metacognition, recognizing the active role of the individual in self-regulating his/her learning process, is strongly correlated with academic success (Magno and Lajom, 2008) – and is surely stimulated by experiences of social interaction (Zimmerman and Schunk, 2011) and cognitive conflict that are typical in many civic and political experiences (Ferreira et al., 2012). Thus, the pedagogical quality of participation experiences might well be highly stimulating to metacognitive activity, and also mirror the metacognitive training developed in schools.

What is at stake in this paper is the pedagogical quality of formal and non-formal educational experiences, as they translate into opportunities for acting and reflecting upon, and during, the experience. To what extent are these experiences bridged, and how does this contribute to metacognition?

## Materials and Methods

In order to investigate how the quality of civic and political experiences relates to dimensions of metacognition we will consider the main predictors of students’ learning strategies, and the relative role of individual characteristics, family cultural background, school variables and civic and political participation in this.

First, based on previous studies showing that gender and age are important variables in explaining metacognitive abilities (Liliana and Lavinia, 2011; Weil et al., 2013), we predict that these individual variables will help explain the variance in metacognition – Hypothesis 1.

Second, considering that school belonging and academic self-efficacy are crucial elements to fully understand academic performance (Pressley, 1986; Neves and Faria, 2006), we predict that academic self-efficacy – Hypothesis 2 – and sense of school belonging – Hypothesis 3 – will play the strongest role in contributing to metacognition.

Third, we expect that civic and political experiences will positively predict metacognition (Hypothesis 4), with high quality experiences predicting critical thinking skills more significantly (Hypothesis 5), as the quality of participation experiences is related with complex political thinking promoted by the confrontation with different points of view (Ferreira et al., 2012).

Forth, despite the fact that previous research has shown a positive association between family variables (parents’ education and books at home) and students’ educational attainment (Dávila and Mora, 2004), we expect that quality of participation, together with civic and political experiences, will exert a stronger predictive power than cultural capital, as other studies indicate that the developmental impact of the quality of participation can go beyond deficits associated with individual background (Fernandes-Jesus et al., 2012) – Hypothesis 6.

### Participants and Procedure

A total of 732 Portuguese students (53.8 % female) from Grades 8 (47.7%, *n* = 349) and 11 (52.3%, *n* = 383) participated in the study. Gender distribution is balanced in the Grade 8 subsample (Female = 173; Male = 176), and less so in Grade 11, with more than half of the sample (57.5%) being females (Female = 221; Male = 162).

Participants were asked to fill out a self-report questionnaire during classes, in schools from the north and center of Portugal, including urban and rural contexts. We obtained parental approval from all under-age participants. The average time needed for filling out the questionnaire was approximately 40 min. For this study, the dependent measures comprise two dimensions of metacognition: cognitive and metacognitive strategies and resource management strategies.

### Measures

#### Metacognition

To investigate the metacognitive learning strategies, we used four sub-scales from the Portuguese version of the Motivated Strategies for Learning Questionnaire (Pintrich et al., 1991; Melo et al., 2006); a 5-point Likert-type scale ranging from 1 (totally disagree) to 5 (totally agree) was used. The scale included the following sub-scales: Elaboration (e.g., “I try to connect ideas of each subject with ideas of other subjects”; four items – Cronbach’s α = 0.81), Critical Thinking (e.g., “I treat the subjects’ material as a starting point and try to develop my own ideas about it”; five items – Cronbach’s α = 0.85), Effort Regulation (e.g., “Even when subjects’ materials are dull and uninteresting, I manage to keep working until I finish”; four items – Cronbach’s α = 0.70), and Peer Learning (e.g., “I try to work with other colleagues, in order to finish my school work”; four items – Cronbach’s α = 0.71). Taking into account our sample size^1^, confirmatory factor analysis showed an acceptable fit of the measurement model of metacognition [*X*^2^(115) = 748.883; *p* ≤ 0.000; *X*^2^/df = 6.512; CFI = 0.905; RMSEA = 0.070; SRMR = 0.0628]. The scales were created with equally weighted items based on the similarity in the magnitudes of the factor loadings (Spector, 1992), a strategy we used for all the scales.

#### Civic and Political Participation Experiences (PCP)

To explore the levels of civic and political participation during the last 12 months we adapted the Portuguese version of the Political Action Scale (Lyons, 2008; Menezes et al., 2012), using seven items (e.g., “attend a public meeting or demonstration dealing with political or social issues”; “do volunteer work”; “wear a bracelet, sign or other symbol to show support for a social or political cause”; “boycott or buy certain products for political, ethical, or environmental reasons”; “link news, music or videos with a social or political content to my contacts”). The youngsters rated the question “Have I done the following activities during the last 12 months?” The response options ranged from 1 (Never) to 5 (Very often). The reliability of the whole scale was α = 0.68.

#### Quality of Participation Experiences (QEP)

The Participation Experiences Questionnaire (QEP) is a self-report measure created by Ferreira and Menezes (2001, Unpublished) that operationalises the theoretical construct underlying the developmental quality of experiences. The first part requires that individuals consider their civic and political experiences in a range of contexts (youth associations, political youth parties, volunteer groups, etc.), identifying their duration (Never; Occasionally; Less than 6 months; 6 months or more). In the second part, individuals assess their most significant experience in terms of the opportunities for action and reflection; in other words, its “potential for engaging in meaningful issues; solving real-life problems; expressing their own views; and, interacting with different others within a context that values pluralism and allows for analyzing the personal meaning of this experience” (Ferreira et al., 2012, p. 601). This second part includes two dimensions: opportunities for action, with four items (e.g., “to participate in activities (such as petitions, protests, parties, meetings, assemblies, debates, public statements, etc.)”; Cronbach’s α = 0.77) and opportunities for reflection, with four items (e.g., “different perspectives were discussed”; “conflicting opinions gave rise to new ways of looking at the issues”; Cronbach’s α = 0.83), using a 5-point Likert-type scale ranging from 1 (not at all) to 5 (very often). The confirmatory factor analysis performed for the whole sample [*X*^2^/df = 5,471; CFI = 0.981, GFI = 0.981; PGFI = 0.436; RMSEA = 0.064; *P*(rmsea <=0.05) = 0.039] corroborates the reliability and validity of the scale, as in other national and international studies (Ferreira et al., 2012; Menezes et al., 2012).

The Quality of Participation Experiences is not given directly by QEP but results from a clustering procedure that “combines both the action and reflection dimension of participation experiences by classifying participants into groups that distinctly articulate both dimensions” (Ferreira et al., 2012, p. 603). Multiple cluster analyses (Hastie et al., 2009) were employed to classify participation experiences on the basis of similarity derived from the scores of QEP subscales. The squared Euclidean distance was used as proximity measure in an agglomerative hierarchical clustering method. Fusions were made by Ward’s method. The number of clusters was determined by dendrogram, implementing the elbow criterion, and the development of error sum of squares. Finally, the *K*-means procedure was implemented for optimizing the cluster solution. *K*-means is a partition based clustering method to minimize the sum of squared error over all clusters. The three adjusted clusters solution explains about 70% of the variance for both the 8th and the 11th grades. Because we are also interested in the ‘non participants,’ we then added the group with “no participation experiences,” which had not been included in the clustering procedure. Therefore, the final variable has four classified groups: No Participation [*N* = 18 (8th grade); *N* = 43 (11th grade)]; low quality of participation [*N* = 82 (8th grade); *N* = 89 (11th grade)]; medium quality of participation [*N* = 130 (8th grade); *N* = 148 (11th grade)]; high quality of participation [*N* = 119 (8th grade); *N* = 103 (11th grade)]. In subsequent analysis we considered only two clusters: low quality [Action (8th grade: x = 1,43; 11th grade: x = 1,64); Reflection (8th grade: x = 1,54; 11th grade: x = 2,05)] and high quality [Action (8th grade: x = 3,64; 11th grade: x = 4,22); Reflection (8th grade: x = 4,13; 11th grade: x = 4,35)].

#### School Variables

As students’ general attitudes towards the school and themselves have a great impact on their thoroughness in study (Weinstein and Palmer, 1990; Neves and Faria, 2006), we included scales on school belonging and academic self-efficacy. Both dimensions were assessed in a 5-point Likert-type scale ranging from 1 (totally disagree) to 5 (totally agree).

Six items were used to tap students’ *sense of school belonging* (“This school means a lot to me” or “I have friends in this school”). Internal consistency was satisfactory, with Cronbach’s α = 0.78.

To investigate students’ *academic self-efficacy*, which includes expectations about their school performance (including exams, study competences and participation in classroom), we translated and adapted the scale created by Smith et al. (1999) using seven items (e.g., “I believe I can develop good study skills”; “I think I will go as far as I like in school”). Internal consistency was good, Cronbach’s α = 0.86.

Additionally, we also considered the *expected level of school attainment* (1 = Basic education; 2 = Secondary education; 3 = Vocational course; 4 = Bachelor; 4 = Master degree; 5 = PhD).

#### Family Variables

Cultural capital was assessed by asking students about their *father’s educational level* – the response scale ranged from 1 (never attended school) to 5 (attended or finished higher education) – and the *number of books at home* (less than 10, between 10 and 100, and more than 100 books). These are good indicators of learning opportunities (Buchmann, 2002), and cultural capital (Lopes et al., 2009), which in turn have a significant effect on civic and political participation (Amadeo et al., 2002; Menezes et al., 2012) and on metacognitive and self-regulatory skills (Lipina and Colombo, 2009).

#### Individual Variables

Regarding the effect of students’ participation on their metacognition, we took *gender* and *age* as individual predictor variables – the former as a dummy-coded variable –, given the variance they usually introduce on participation and metacognition levels.

### Data Analysis Procedures

#### Linear Regressions

To grasp the predictive effect of combining in- and out-of-school experiences on metacognitive dimensions, we performed linear regressions with the following predictors organized in blocks:

(a) Age, gender;
(b) Expected level of school attainment, parents’ educational level and books at home;
(c) School belonging and academic self-efficacy;
(d) Experiences of civic and political participation (PCP) and quality of participation experiences (QEP).

## Results

**Table 1** presents the model summary for linear regression predicting elaboration. The percentage of variance explained is 42% (**Table 1**). Gender and age explain 2% of the variance, a value that rises to 15% when considering family cultural background factors (books at home, father’s level of education and expected level of school attainment). Tolerance values are always high (>0.10) therefore multicolinearity among predictors does not appear to be a problem. School variables play a very significant role, which slightly increases (around 4%) when including out-of-school civic and political experiences. The major significant predictor is academic achievement, followed by civic and political participation and high quality experiences, and finally gender (female), expected level of school attainment and school belonging (**Table 2**).

**Table 1:** Model summary for linear regressions on elaboration.

Model	*R*	*R* Square	Adjusted *R* Square	Standard Error of the estimate	Change statistics				
					*R* square change	*F* change	df1	df2	Significance *F* change
1	0,152^a^	0,023	0,020	0,80037	0,023	8,362	2	704	0,000
2	0,381^b^	0,145	0,139	0,75040	0,122	33,295	3	701	0,000
3	0,625^c^	0,391	0,384	0,63444	0,246	140,828	2	699	0,000
4	0,655^d^	0,429	0,420	0,61562	0,038	15,468	3	696	0,000

^a^*Predictors: (Constant), sex_male, age*.

^b^*Predictors: (Constant), a + books at home, expected level of school attainment, father’s level of education*.

^c^*Predictors: (Constant), a + b + school belonging, academic self-efficacy*.

^d^*Predictors: (Constant), a + b + c + PCP, low quality experiences, high quality experiences*.

**Table 2:** Regression coefficients on elaboration.

Model	Unstandardized		Standardized			95,0% Confidence		Collinearity
	coefficients		coefficients			interval for B		statistics
	β	St Error	β	*t*	Significance	Lower bound	Upper bound	Tolerance
4 (Constant)				0,332	0,740	-0,540	0,759	
Age	0,016	0,014	0,035	1,156	0,248	-0,012	0,044	0,893
sex_male	-0,107	0,047	-0,066	-2,260	*0,024*	-0,201	-0,014	0,956
books at home	0,049	0,026	0,064	1,893	0,059	-0,002	0,099	0,717
Expected level of school attainment	0,045	0,021	0,072	2,175	*0,030*	0,004	0,086	0,750
Father’s level of education	-0,037	0,027	-0,047	-1,374	0,170	-0,089	0,016	0,706
School belonging	0,086	0,041	0,067	2,095	*0,037*	0,005	0,167	0,799
Academic self-efficacy	0,612	0,041	0,509	15,033	*0,000*	0,532	0,692	0,717
PCP	0,143	0,038	0,117	3,789	*0,000*	0,069	0,218	0,859
Low quality experiences	-0,097	0,060	-0,051	-1,597	0,111	-0,215	0,022	0,820
High quality experiences	0,169	0,057	0,097	2,996	*0,003*	0,058	280	0,791

*Statistically significant values in italic*.

The percentage of variance explained by the linear regression model on critical thinking is 38% (**Table 3**). Tolerance values are always high (>0.10) therefore multicolinearity among predictors does not appear to be a problem. Again, school level variables play an important predictive role, but so do civic and political experiences (**Table 4**). The significant predictors are academic self-efficacy, followed by high quality experiences and civic and political participation, and finally gender (male) and age (negatively).

**Table 3:** Model summary for linear regressions on critical thinking.

Model	*R*	*R* square	Adjusted *R* square	Standard error of the estimate	Change statistics				
					*R* square change	*F* change	df1	df2	Significance *F* Change
1	0,158^a^	0,025	0,022	0,80726	0,025	8,999	2	703	0,000
2	0,329^b^	0,108	0,102	0,77363	0,083	21,817	3	700	0,000
3	0,565^c^	0,319	0,313	0,67687	0,211	108,218	2	698	0,000
4	0,618^d^	0,381	0,372	0,64669	0,062	23,220	3	695	0,000

^a^*Predictors: (Constant), sex_male, age*.

^b^*Predictors: (Constant), a + books at home, expected level of school attainment, father’s level of education*.

^c^*Predictors: (Constant), a + b + school belonging, academic self-efficacy*.

^d^*Predictors: (Constant), a + b + c + PCP, low quality experiences, high quality experiences*.

**Table 4:** Regression coefficients on critical thinking.

Model	Unstandardized		Standardized			95,0% Confidence		Collinearity
	coefficients		coefficients			interval for B		statistics
	β	Standard error	β	*t*	Significance	Lower bound	Upper bound	Tolerance
4 (Constant)				2,783	0,006	0,285	1,650	
Age	-0,031	0,015	-0,066	-2,094	*0,037*	-0,061	-0,002	0,893
Sex_male	-0,105	0,050	0,064	2,101	*0,036*	0,007	0,203	0,957
Books at home	0,010	0,027	0,013	0,382	0,702	-0,043	0,063	0,718
Expected level of school attainment	0,029	0,022	0,046	1,330	0,184	-0,014	0,072	0,750
Father’s level of education	-0,004	0,028	-0,005	-0,142	0,887	-0,059	0,051	0,705
School belonging	0,035	0,043	0,027	0,817	0,414	-0,050	0,120	0,801
Academic self-efficacy	0,573	0,043	0,472	13,400	*0,000*	0,489	0,657	0,719
PCP	0,150	0,040	0,121	3,764	*0,000*	0,072	0,228	0,859
Low quality experiences	-0,094	0,063	-0,049	-1,486	0,138	-0,219	0,030	0,820
High quality experiences	0,287	0,060	0,162	4,820	*0,000*	0,170	0,404	0,790

*Statistically significant values in italic*.

The model summary for peer learning (**Table 5**) explains 26% of the variance. Tolerance values are always high (>0.10), therefore multicolinearity among predictors does not appear to be a problem. Although the percentages of variance explained are lower than in the previous metacognitive dimensions, in peer learning both school and out-of-school variables seems to play an important role. The significant predictors are academic self-efficacy, the quality of participation (positively, when high, negatively, when low), civic and political participation, gender (female), school belonging and books at home (negatively; **Table 6**).

**Table 5:** Model summary for linear regressions on peer-learning.

Model	*R*	*R* square	Adjusted *R* square	Standard error of the estimate	Change statistics				
					*R* square change	*F* change	df1	df2	Significance *F* Change
1	0,180^a^	0,033	0,030	0,80005	0,033	11,826	2	703	0,000
2	0,249^b^	0,062	0,055	0,78955	0,029	7,275	3	700	0,000
3	0,434^c^	0,188	0,180	0,73549	0,126	54,344	2	698	0,000
4	0,509^d^	0,259	0,249	0,70406	0,071	22,240	3	695	0,000

^a^*Predictors: (Constant), sex_male, age*.

^b^*Predictors: (Constant), a + books at home, expected level of school attainment, father’s level of education*.

^c^*Predictors: (Constant), a + b + school belonging, academic self-efficacy*.

^d^*Predictors: (Constant), a + b + c + PCP, low quality experiences, high quality experiences*.

**Table 6:** Regression coefficients on peer-learning.

Model	Unstandardized		Standardized			95,0% Confidence		Collinearity
	coefficients		coefficients			interval for B		statistics
	β	Standard error	β	*t*	Significance	Lower bound	Upper bound	Tolerance
4 (Constant)				3,464	0,001	0,568	2,054	
Age	-0,004	0,016	-0,009	-0,273	0,785	-0,036	0,028	0,893
Sex_male	-0,167	0,054	-0,103	-3,076	*0,002*	-0,274	-0,060	0,957
Books at home	-0,058	0,029	-0,076	-1,963	*0,050*	-0,116	0,000	0,718
Expected level of school attainment	0,034	0,024	0,054	1,440	0,150	-0,012	0,081	0,750
Father’s level of education	-0,041	0,031	-0,052	-01,340	0,181	-0,101	0,019	0,705
School belonging	0,141	0,047	0,109	2,987	*0,003*	0,048	0,233	0,801
Academic self-efficacy	0,372	0,047	0,308	7,995	*0,000*	0,281	0,464	0,719
PCP	0,159	0,043	0,130	3,684	*0,000*	0,074	0,244	0,859
Low quality experiences	-0,150	0,069	-0,078	-2,174	*0,030*	-0,286	-0,015	0,820
High quality experiences	0,274	0,065	0,155	4,225	*0,000*	0,147	0,401	0,790

*Statistically significant values in italic*.

Finally, this model does not explain a relevant percentage of variance for effort regulation (7,3%).

## Discussion

Our results suggest that youth experiences in both schools and communities promote important learning competences. The articulation between these two pedagogical spheres enables grasping the bigger picture of the learning process, valuing experiences inside and outside the school, and suggesting that instructional strategies should acknowledge the pedagogical value of civic and political experiences that can stimulate crucial competences for academic performance.

Concerning the influence of gender in explaining metacognition, our results seem to echo the argument about the ambivalent role of gender in self-regulation (Bidjerano, 2005), as our results favor female students regarding elaboration and peer-learning, but indicate the positive influence of being male on critical thinking. Therefore, despite the fact that results are ambivalent, gender plays a role in predicting metacognition (Hypothesis 1). With regard to age, the results do not corroborate the research indicating that metacognition improve with age (Weil et al., 2013): in our study, age does not significantly explain variances in students’ metacognition, exception made to critical thinking, in which the younger group (8th grade) is more likely to make critical evaluations of ideas and academic content (Hypothesis 1).

Although school belonging, an important variable in explaining academic achievement (Moallem, 2013), predicts peer-learning and elaboration, its role is always less significant than civic and political participation (not confirming the Hypothesis 3). As expected, academic self-efficacy has the highest predictive power in explaining variances in students’ metacognition (supporting Hypothesis 2). Civic and political variables generally come up second to academic self-efficacy, be it when we consider high quality experiences (Hypothesis 5) or the involvement in civic and political activities (Hypothesis 4). The developmental quality of participation seems to make the difference (compared to mere civic and political engagement) regarding peer-learning, and particularly critical thinking (consistent with Hypothesis 5).

The linear regression model unveils a weak association between our model (which combines participation and schooling variables) and resources management strategies, particularly regarding effort regulation (explaining only 7,3% of its variance), and peer-learning (explaining just 26% of its variance). Still, the unusual combination of variables in our model explains a considerable percentage of variance in metacognition, with civic and political experiences (including quality of participation) predicting almost all dimensions, even more than cultural capital variables (parents’ level of education and books at home) – supporting Hypothesis 6. Furthermore, when analyzing metacognition skills, the influence of meaningful civic and political experiences that promote opportunities for reflection in supportive and challenging relational contexts seems to transcend the role of the sense of school belonging, which, while being also a contextual variable, is nonetheless related to the meaning individual’s attribute to school – this is particularly clear regarding critical thinking, in which the quality of participation exerts a strong predictive power, while the sense of school belonging is not a significant predictor. Our results highlight the relevance of combining academic self-efficacy – an individual variable related with the level of engagement and commitment with schooling – with civic and political life experiences. Additionally, it is worth noting that the developmental quality of participation plays a major role in explaining students’ metacognition levels – generally in addition to simple experiences of civic and political participation.

Indeed, experiences of civic and political participation may have an important role mainly in fostering the ability to link and transfer concepts, and to building a learning process based on questioning and critical thinking (Hypothesis 4 and 5). The model shows that metacognition is also stimulated by experiences favoring cognitive conflict and social perspective taking, as happens in civic and political participation experiences with high developmental quality. This is consistent with previous research that demonstrates the positive effects of high quality participation experiences at the individual level (Ferreira et al., 2012). These results suggest that this line of research does have potential and deserves further exploration. Previous studies already show that academic performance can be improved by students participation in sports (Khan et al., 2012), in policy debate programs (Mezuk et al., 2011), in extra-curricular activities (Roberts, 2007, Unpublished), or in community service (Schmidt et al., 2007). Our results go beyond school-based participation experiences, showing the role of out-of-school civic and political participation (particularly if combined with academic self-efficacy) in metacognition.

By adding the cognitive processes involved in school performance, this research bridges the research gap in the relationship between cognitive processes involved in civic and political participation (the cognitive conflict, crucial to cognitive development, which is present in contexts promoting quality of participation) and those that contribute to educational success (metacognition). In order to be self-regulated, students should be active participants in their own learning, knowing how to think and how to adapt and modify learning strategies in order to achieve their academic goals. In the same way, for civic and political participation to be considered meaningful and transformative, it must entail balanced opportunities for action and reflection in a supportive context, facilitating cognitive conflict in order to promote development. Lamm (2009, p. 92) already suggests a similar connection when she argues that self-regulated learning should be linked to political engagement, stating that “students should be provided with opportunities to connect the attributes they are learning with real-world opportunities to practice them […] students need to be metacognitive about those attributes, which is referred to as self-regulated learning.” The results presented in this paper corroborate the relevance and need for such a connection; additionally, they show that civic and political experiences (particularly when they are personally meaningful, in terms of developmental quality) can also play an important role in fostering important competences for school success.

Annette (2006), discussing the application of Kolb’s pedagogy of experiential learning to citizenship education, emphasizes that students learn not just through volunteering and civic engagement, but through reflection on their experience. By giving due consideration to the political realm, this study adds to the existing literature. Indeed, just like the pedagogical efforts in developing experiential learning as a way of improving academic performance have yet to “go beyond traditional volunteering and doing good works and link the service learning with political knowledge, skills, and understanding” (Annette, 2006, p. 1), research on academic performance, while dealing extensively with the impacts of civic participation – particularly school-based participation –, falls short of considering the specific impacts of political participation. It appears, then, that there is a widespread preference for the non-political. This, of course, is in itself a revealing choice, as the presumably more sensitive, tricky field of politics is left outside the radar of intervention and analysis. Therefore, we agree with Cress et al. (2010) when they argue that there is a “promising connection” between civic engagement and academic success, one which deserves further attention. In line with these authors, we strongly believe in the importance of bridging in-and out-of-school learning. In fact, we know, since Dewey (1916), that in order to increase the individual and collective relevance of learning, the school must be connected to the community, which ultimately leads us to the relevance of considering the quality of participation experiences.

## Conclusion

By articulating the field of metacognition with civic and political participation this paper brings together two research domains that have, as yet, been mostly estranged from each other. We hope to have shown that there are relevant connections between them and, thus, hope to be contributing to this emerging field. In particular, we would like to emphasize that the relationship between the quality of participation experiences and the metacognitive learning strategies of a more dialectical and conflictual nature (elaboration and critical thinking) seems to provide added strength to the pertinence of the study of this topic, namely because there are clear educational implications stemming from it. Indeed, improvements in learning may result from a better articulation between the school and civic and political participation. Educational agents need to recognize this linkage. Further research is needed in order to clearly understand the impact of civic and political variables on academic success: (a) eventually establishing causality relations between civic and political behaviors and self-regulated learning; (b) determining what are the contexts and forms of participation that impact more strongly on self-regulated learning; and (c) assessing how the pedagogical value of civic and political experiences can compensate for the negative influence that cultural, economic and social disadvantages have on school performance. Longitudinal studies could be of added value in understanding these links. Our study participates in and contributes to this debate, demonstrating the pertinence of such questions.

Acknowledging the pedagogical value of civic and political participation, namely high quality experiences, equates to acknowledging the permeability of formal and non-formal educational contexts. Ultimately, then, this means acknowledging that there is a link between the definition and development of democratic citizenship and the choices made regarding modes of teaching and learning, and indeed the very configuration of the educational system. Disciplines such as history and sociology have pointed to this relationship from their particular viewpoints (Zeigler and Peak, 1970; Benavot, 1996). Here we provide different, added strength to this thesis by demonstrating its validity from an interdisciplinary viewpoint.

## Author Contributions

The CM conducted the study as part of her PhD, under the supervision of the TN and IM, and wrote the main part of the paper, again the TN and IM contributions and revisions. PT was involved in data analysis and presentation.

## Conflict of Interest Statement

The authors declare that the research was conducted in the absence of any commercial or financial relationships that could be construed as a potential conflict of interest.

## Footnotes

1 Confirmatory factor analysis and other procedures regarding the dimensionality of the questionnaires were performed on a larger sample of 1107 students that includes a sub-sample of higher education students.
